# Radiating on Oceanic Islands: Patterns and Processes of Speciation in the Land Snail Genus *Theba* (Risso 1826)

**DOI:** 10.1371/journal.pone.0034339

**Published:** 2012-04-06

**Authors:** Carola Greve, France Gimnich, Rainer Hutterer, Bernhard Misof, Martin Haase

**Affiliations:** 1 Zoologisches Forschungsmuseum Alexander Koenig, Bonn, Germany; 2 Vogelwarte, Zoologisches Institut, Ernst-Moritz-Arndt-Universität Greifswald, Greifswald, Germany; University of Texas, United States of America

## Abstract

Island radiations have played a major role in shaping our current understanding of allopatric, sympatric and parapatric speciation. However, the fact that species divergence correlates with island size emphasizes the importance of geographic isolation (allopatry) in speciation. Based on molecular and morphological data, we investigated the diversification of the land snail genus *Theba* on the two Canary Islands of Lanzarote and Fuerteventura. Due to the geological history of both islands, this study system provides ideal conditions to investigate the interplay of biogeography, dispersal ability and differentiation in generating species diversity. Our analyses demonstrated extensive cryptic diversification of *Theba* on these islands, probably driven mainly by non-adaptive allopatric differentiation and secondary gene flow. In a few cases, we observed a complete absence of gene flow among sympatrically distributed forms suggesting an advanced stage of speciation. On the Jandía peninsula genome scans suggested genotype-environment associations and potentially adaptive diversification of two closely related *Theba* species to different ecological environments. We found support for the idea that genetic differentiation was enhanced by divergent selection in different environments. The diversification of *Theba* on both islands is therefore best explained by a mixture of non-adaptive and adaptive speciation, promoted by ecological and geomorphological factors.

## Introduction

A major problem for the study of speciation is that the formation of new and genetically isolated species is in most cases a slow and continuous process, lasting many generations. The direct observation of the entire process of speciation is usually impossible, except in the case of polyploid or hybrid speciation, which can occur in one or a few generations [1]. Speciation has yet to be studied by comparing many snap-shots of this continuous process - from the divergence of populations to fully reproductively isolated species [2]. Compared to continental regimes, islands provide isolated systems often with less complex biotas, in which speciation processes can be studied more effectively. Consequently, island systems have been successfully used to study adaptive diversification of ecologically and/or morphologically well differentiated sympatric species as well as non-adaptive (cryptic) radiations of ecologically or morphologically similar allopatric or parapatric species [3]–[8]. The study of island radiations have thus helped to establish our current understanding of allopatric, sympatric and parapatric speciation, but the relative importance of these three modes is still unclear. In this context, it is remarkable that species divergence often correlates with island size. This phenomenon has been interpreted as evidence for the important role of geographic isolation (allopatry) in speciation [7].

Here, we analyzed the divergence of the land snail genus *Theba* [9] on two of the Canary Islands: Lanzarote and Fuerteventura. *Theba* populations on these two islands are found in allopatry, parapatry and sympatry, displaying different levels of molecular and morphological differentiation [10]. The geological characteristics of Lanzarote and Fuerteventura, including unusual longevity, complex volcanic evolution and close vicinity to a continental land mass [11] provide ideal conditions to study the interplay of biogeography, dispersal ability and differentiation in generating species diversity.

The volcanic archipelago of the Canary Islands is located in the east Atlantic Ocean with Fuerteventura lying approximately 100 km off the West Saharan coast of NW Africa. The archipelago has never been connected to continental landmasses. The eastern-most islands, Lanzarote and Fuerteventura, emerged 24 million years ago (mya) and form one large volcanic edifice separated by shallow waters less than 50 m deep. During periods of glaciation and low sea level in the Quaternary, they were periodically connected by land bridges [12]–[14]. Due to intense erosion, Lanzarote and Fuerteventura lack the high elevations seen on other Canary Islands, which are exposed to the humid north-eastern trade winds. Both islands are characterized by an arid climate, enhanced by the continental influence of the Saharan desert, and are dominated by xeric scrub vegetation. Local hygrophilous fauna and flora can be found only atop a few mountains (Jandía on Fuerteventura and Famara on Lanzarote) [11]. Though the eastern islands exhibit less diverse habitat structure than the central and western Canary Islands, they are characterized by complex geomorphology due to past and recent volcanic and erosional activity [11]–[12].

Despite the close proximity of NW Africa to the Canary Islands, the main speciation mode of the genus *Theba* on the islands has been intra-archipelago diversification, rather than independent colonization of the islands from the African continent [10]. The phylogenetic study of Greve et al. [10] was based on COI and ITS1 sequence data and indicated that differentiation within *Theba* species on Fuerteventura and Lanzarote is more extensive than previously thought [15]–[17]. These molecular data helped to identify three endemic species on Lanzarote and two on Fuerteventura. Neither the species from Lanzarote nor those from Fuerteventura appear to be monophyletic. These results, however, were based on sequence information of only a few specimens. In order to assess the extent and nature of population differentiation within and between the *Theba* species of Lanzarote and Fuerteventura, we expand existing COI sequence data and additionally conducted comprehensive morphological and population genetic (AFLP) analyses. As a null hypothesis, we assumed that the long and dynamic history of both islands and the relatively uniform ecological situation along with the low dispersal abilities of land snails, led to isolation by distance (IBD) and eventually allopatric speciation within and among islands. The greater habitat diversity on the southern Jandía peninsula of Fuerteventura [11], however, might have supported ecological (adaptive) diversification. Therefore, we sampled populations extensively along a north-south transect on both islands hopefully covering different levels of genetic differentiation. Additionally, we sampled specimens from different ecological habitats on the Jandía peninsula. We then used a population genomic approach based on the AFLP data to differentiate between different modes of speciation within and among both islands, under the assumption that allopatric speciation leads to random distribution of differentiation within the genome, whereas sympatric/parapatric speciation leads to a specific non-random differentiation of alleles due to divergent selection [18]–[19].

## Materials and Methods

### Sampling and DNA isolation

**Figure 1:**
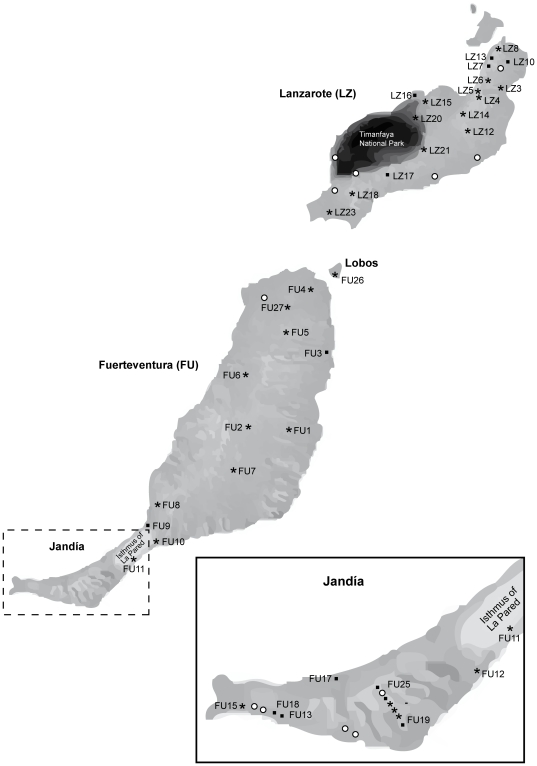
Sampling localities of specimens of *Theba* on Lanzarote and Fuerteventua. The Jandía peninsula is enlarged for better recognition. * = ∼20 specimens collected, ▪ = fewer than 20 specimens collected, ○ = empty shells.

Based on the preliminary results of Greve et al. [10], we sampled *Theba* populations extensively on Fuerteventura and Lanzarote. Snails were collected on these islands in December 2009. In total, forty-one populations were sampled along a north-south transect, covering all known genetically distinguishable clades of *Theba* on Fuerteventura and Lanzarote [10] (Fig. 1). In order to cover the entire extent of differentiation, we included all autochthonous species from the Canary Islands, viz. *T.* cf. *arinagae* and *T. grasseti* from Gran Canaria and *T. macandrewiana* from the Selvagens Islands (Table S1). *T. macandrewiana* was used as the outgroup species. Snails were preserved in absolute ethanol and total genomic DNA was extracted from foot muscle tissue of each snail using the DNeasy® Blood & Tissue Kit (Qiagen) following the manufacturer's protocol. Vouchers are available at the Zoologisches Forschungsmuseum Alexander Koenig, Bonn, Germany.

### Mitochondrial DNA sequencing

At least two specimens per population were sequenced for a fragment of the mitochondrial cytochrome *c* oxidase subunit I (COI). The COI fragment was amplified by the polymerase chain reaction (PCR) using the primer combination LCO-1490 [5′-GGTCAACAAATCATAAAGATATTGG-3′ [20]] and C1-N-2191 [5′-CCCGGTAAAATTAAAATATAAACTTC-3′ [21]]. PCR reactions were carried out in a total volume of 10 µL using the Qiagen Multiplex PCR Kit. Thermal cycling conditions were as follows: 95°C for 15 min, 15 cycles of touchdown PCR (94°C for 35 s, 55°C–40°C annealing for 90 s and 72°C extension for 90 s) followed by 25 cycles (94°C for 35 s, 40°C annealing for 90 s and 72°C extension for 90 s) and a final extension step at 72°C for 10 min. PCR products were purified using ExoSAP-IT® (USB). Double stranded sequencing was carried out by a sequencing facility (Macrogen, Seoul, South Korea; using ABI 3730XL sequencers). Sequences were deposited in GenBank (see Table S1).

### AFLP genotyping

The variation of standard nuclear genes in pulmonates does not provide enough resolution to sort taxa below genus level [22]. We therefore used the amplified fragment length polymorphism (AFLP) method to control for potential phylogenetically misleading lineage sorting of mtDNA. If possible, we sampled 20 individuals per population to achieve accurate results for estimating population structure in the AFLP data [23].

AFLP markers were obtained with a slightly modified version of the original protocol of Vos et al. [24]. Selective amplifications were performed using six different primer combinations: *Eco*RI-ACA/*Mse*I-CTG, *Eco*RI-ACA/*Mse*I-CTT, *Eco*RI-ACC/*Mse*I-CAC, *Eco*RI-AGG/*Mse*I-CTG, *Eco*RI-AGG/*Mse*I-CTC and *Eco*RI-ACT/*Mse*I-CAG. The fluorescently labeled fragments were separated by electrophoresis on a CEQ™ 8800 capillary sequencer (Beckman Coulter, Inc., Fullerton, California), with an internal size standard (CEQ DNA Size Standard Kit 600, Beckman Coulter, Inc.). Signal detection, processing and binning of the AFLP electropherograms were carried out using the CEQ™ System Fragment Analysis module of the manufacturer's software (Version 9.0.25, Beckman Coulter, Inc.). The fluorescence threshold for an accepted signal was set to 1% of the height of the second largest peak detected in the AFLP profile. Choosing a relative threshold instead of the frequently used fixed threshold minimizes artifacts resulting from differences in total profile strength among individuals as well as those resulting from unequal detection among capillaries [25]. Correct fit of the size standard and fragment distribution was checked for all profiles. Low quality profiles were discarded. Subsequently, fixed fragment categories (hereafter also referred to as bins) were created between 60 and 550 bases (b). AFLP markers were automatically scored according to the presence/absence of fragment peaks within each bin and for each sample, setting the fluorescent signal detection threshold to 50 units. According to the accuracy of the CEQ sequencing system (standard deviation = 0.25 b; manufacturer's specifications), the maximum bin width for reliable fragment sizing was set to 0.75 b. Monomorphic markers were excluded from the data set.

To ensure high reliability of AFLP genotyping, 11% of the samples were genotyped twice for all primer combinations; these replicates were taxonomically representative of the whole data set. A perl script was written to fully automate the following marker selection procedure and to estimate the average genotyping error rate per marker (following [26]–[27]). Based on the replicated samples, the repeatability of each individual marker was estimated to control for scoring errors. Bins with less than 81% repeatable markers were excluded from the data set. Furthermore, all bins without any fragment peak present among replicates were excluded, because shared fragment absences (null alleles) are particularly prone to homoplasy due to the multiple and independent ways in which a fragment can be lost [28]–[29]. Bins without any confirmed (present/present) fragment peak among compared replicate pairs were also excluded to avoid spurious background noise in the data set. Finally, the remaining markers were used to estimate the average genotyping error rate per marker. This value was 7.6% and was lower than the maximum value of 10% recommended by Bonin et al. [23]. The final AFLP binary character matrix including all replicates is provided as supporting information (Dataset S1).

### Phylogenetic analyses

COI sequences were aligned with ClustalW [30] using default parameter settings and obviously misaligned positions were adjusted manually in Bioedit v7.0 [31]. Homogeneity of base frequencies among COI sequences was checked with the χ^2^ - test implemented in PAUP* v4.0b10 [32]. For phylogenetic reconstruction Bayesian (BA) and maximum likelihood (ML) analyses were performed. According to the results of the Akaike Information Criterion in MrModeltest v2.3 [33], the GTR+Γ+I model was selected for BA. BA was carried out with MrBayes v3.1 [34]–[35] using two parallel runs each with 6 simultaneous Markov chains for 25,000,000 generations. Trees were sampled every 100th generation. Excluding the first 120,000 trees of each run as burn-in, a 50% majority-rule consensus tree with posterior probabilities was constructed from the remaining 260,002 trees. Tracer v1.4.1 [36] was used to determine the burn-in generation number as well as to check convergence of parameter estimates by inspecting effective sample size (ESS) values and traces of the MCMC samples. ML analysis was conducted with RAxML v7.0.3 [37] using the GTR+Γ+I model. Node support for the best-scoring ML tree was evaluated with 1000 rapid bootstrap replicates [38].

Phylogenetic reconstruction based on the AFLP data set was performed with PAUP* v4.0b10 [32] using neighbor-joining (NJ) on Nei-Li [39] distances. This distance measure is best suited for AFLPs, as it accounts for the sharing of presence alleles, while absent alleles are ignored due to their more homoplasious character [28]. Internal node support was assessed using nonparametric bootstrapping (1000 replicates).

### MOTU assignment

We considered the criterion of reciprocal monophyly to infer possible boundaries of molecular operational taxonomic units (MOTU). We further tested accurate MOTU assignments among specimens of the COI data set by using the cluster module of TaxonDNA v.1.7.8 [40]. This module groups sequences at different similarity thresholds into clusters based on pairwise uncorrected p-distances. We chose a clustering threshold of 3%, as this threshold has been cited as sufficient genetic disparity for species delimitation [41].

### Morphometrics

Samples used for morphometrics largely consisted of empty shells (Table S1). However, only in the cases of the taxonomically unproblematic flat shelled *T. grasseti* and *T. impugnata* did we include samples from localities that remained genetically uninvestigated.

**Figure 2:**
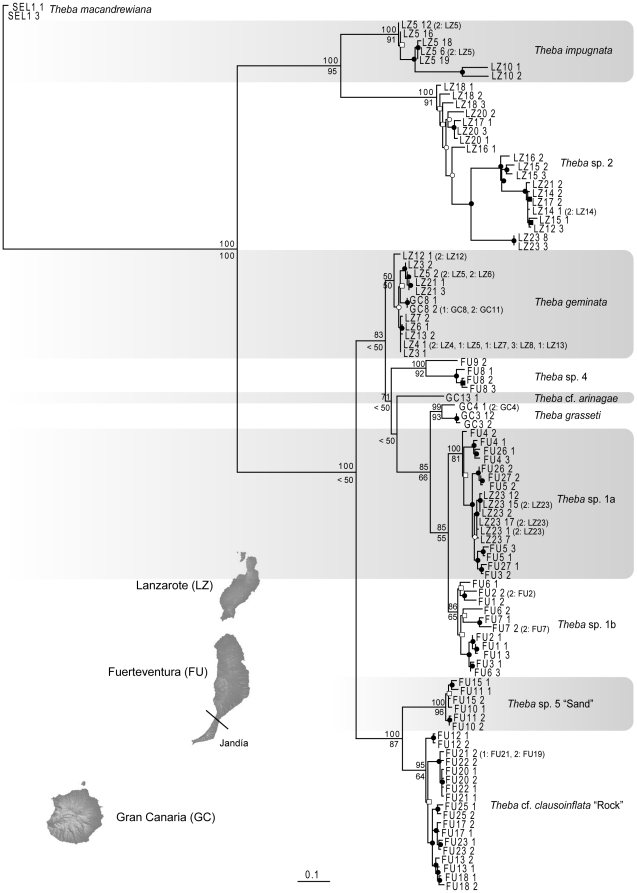
RAxML tree based on partial COI sequences of *Theba* sampled on the Canary Islands. Bootstrap support (BS) values (1000 replicates) of the ML run are indicated below branches. Numbers above branches refer to Bayesian posterior probabilities (BPP) of the Bayesian analysis (BA). ▪ = BS support >50%, no BPP support; □ = BS support <50%, no BPP support; • = BS support and BPP >50%; ○ = BS support <50% and BPP >50%.

**Figure 3:**
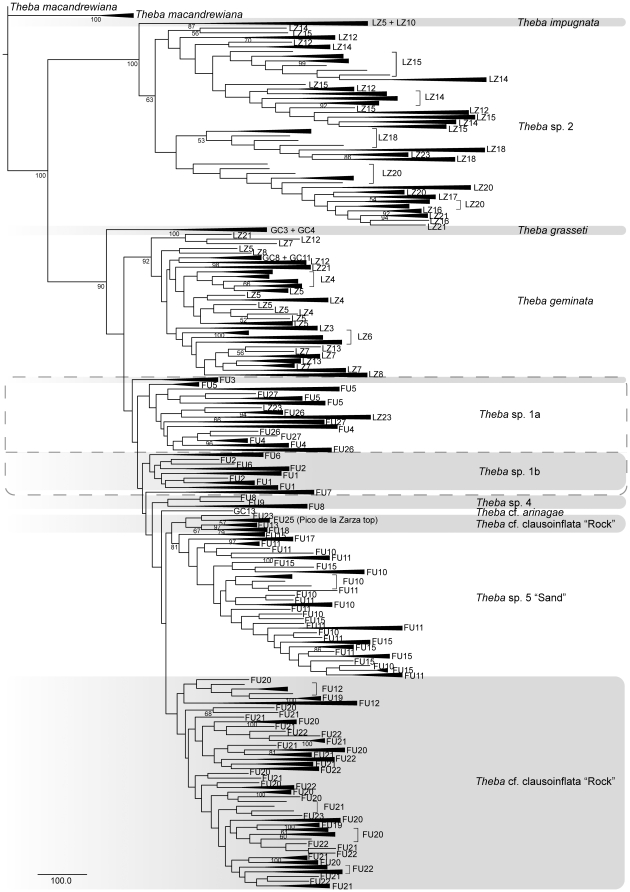
AFLP neighbor-joining (NJ) tree of Canary Islands *Theba* based on Nei-Li distances. The AFLP data set consisted of 1964 loci. Bootstrap support values (1000 replicates) are indicated below branches.

Shell morphology was analyzed using geometric morphometrics [42]–[43]. Shells were balanced on a base of Styrofoam and the apertural view photographed at the same scale using a Nikon D-70s camera equipped with an AF-Nikkor 28–105 mm lens. Ten landmarks were applied using tpsDig [44] after the images had been transformed into tps format in tpsUtil [45]. The procedure has proved to be highly repeatable for depressed, globular and conical shells as well as for shells of both coiling directions [46]–[47]. Multivariate statistical analyses were carried out based on Procrustes superimpositions using the programs of the IMP suite of H.D. Sheets and co-workers (http://www3.Canisius.edu/~sheets/morphsoft.html) and PAST 2.0 [48]. Centroid-size (square root of the summed squared distances of each landmark form the centroid of the landmark configuration [43]) was used as a proxy for shell size. Morphometric comparisons were based on MOTUs defined by the phylogenetic analyses (Figs. 2, 3).

Single population samples were treated as units only if they were apparently morphologically different, for example FU23 and FU25. Samples composed of two species (hereafter referred to as “mixed samples”) were only used in morphological assignment tests based on foregoing CVAs (see manual to CVAgen6 of the IMP suite) to estimate the proportion of each species involved. Similarly, FU17 was only used in a morphological assignment test because of its ambiguous topological position. Except for the genetically identified individuals of LZ23, shells from mixed samples were not included in the overall analysis based on the results of the morphological assignment tests, because of the high rate of incorrect allocations.

### Population structure based on the AFLP data set

We used Population graphs, a multivariate graph theoretical approach [49], to examine the genetic structure among populations. This method is free of an *a priori* model of population arrangement, unlike AMOVA, and generates a graph describing the high-dimensional genetic covariance relationships among all populations simultaneously. The Population graph contains the minimal number of edges that sufficiently describe the among population genetic covariance structure, with node size representing the within population genetic variance and edge lengths representing the among population component of genetic variation. As the sample size cut-off value per population is three for Population graphs, populations with fewer than three individuals were excluded from the analysis (Table S1).

We also used *Structure* v2.3.2 [50]–[51] to investigate patterns of genetic structure. Analyses were conducted without *a priori* group designation using a model allowing for recessive alleles, which is best suited for dominant molecular markers such as AFLPs [51]. We chose an admixture model with correlated allele-frequencies [52]. We allowed for gene flow, thus avoiding inaccurate presumptions about genetic barriers. The dirichlet parameter for the degree of admixture (α) and the parameter of allelic frequencies distribution (λ) were set to be inferred from the data. For all *Structure* analyses, we used a total run length of 250,000 generations, including a burn-in of 50,000 generations. According to the sampled populations, *K* = 1 to 46 was tested with ten independent runs at each *K* ( = number of populations or clusters). We plotted the mean likelihood *L*(*K*) over 10 runs for each *K* and used the statistic Δ*K* proposed by Evanno et al. [53] to determine the optimal number of genetically differentiated clusters.

Genetic structure of the AFLP data was further investigated using analysis of molecular variance (AMOVA) as implemented in Arlequin v3.5 [54]. For this analysis, all populations with fewer than 5 individuals were excluded (Table S1). AMOVA was based on unstructured as well as several subdivided data sets. Hierarchical levels comprised within- and between-group comparisons (Table S2) according to geography and MOTUs. Significance of variance components was tested with 20022 permutations.

### Isolation by distance

To test for IBD, a simple Mantel test [55] was conducted using the software zt [56]. The significance of the test was achieved by permuting the matrices 1,000,000 times. Matrices of pairwise F_ST_ values for all populations of Lanzarote and Fuerteventura were calculated using Arlequin v3.5 [54]. Geographical distances (in kilometers) between sample sites were computed with AFLPdat [57]. All Mantel tests were based on the AFLP data set.

### Outlier detection

Outlier detection among AFLPs was performed with BayeScan [58] using default parameter settings. It is based on the idea that genetic differentiation among populations in contrasting environments is expected to be different for loci under selection than for the rest of the genome. BayeScan estimates a posterior probability for each locus being under selection. All loci with a posterior probability over 95% were retained as outliers. Compared to alternative programs, BayeScan is less sensitive to false-positive outlier detection and allows for different demographic histories and different amounts of genetic drift between the populations [59]–[60]. Inter- and intraspecific pairwise comparisons between populations occurring in allopatry, parapatry and sympatry were used to infer mechanisms of ongoing and past speciation [18]–[19]. The pairwise analyses allow the identification of loci that are outliers in multiple population pairs of compared MOTUs. Only outlier loci found in every population pair of compared MOTUs (hereafter referred to as consistent outlier loci) were considered as candidate markers most likely under selection, thus reducing type I errors [61]. Following Butlin [1], we refrained from a categorization of loci into outlier and non-outlier groups.

## Results

### Phylogenetic analyses

In total, 125 specimens were sequenced for COI resulting in a data set of 606 aligned positions. This included the 11 COI sequences from the previously published data set [10]. Base composition among sequences was homogeneous (χ^2^ = 127.11, d.f. = 372, p>0.999). Phylogenies obtained from ML and BA analyses were highly consistent, with only slight topological differences (Fig. 2). Both approaches supported ten MOTUs on the eastern Canary Islands (see the results of the MOTU assignment test). In the phylogenetic tree, *T. impugnata* and *Theba* sp. 2 from Lanzarote formed a monophyletic group (Bayesian posterior probability (BPP) = 100%; bootstrap (BS) = 95%) and were sister to all other MOTUs (BPP = 100%; BS = 100%). The monophyletic group of the remaining MOTUs (BPP = 100%; BS<50%) fell into two subgroups. One subgroup (BPP = 100%; BS = 87%) comprised all populations of the Jandía peninsula on Fuerteventura with *Theba* cf. *clausoinflata* “Rock” sister to *Theba* sp. 5 “Sand”. The other subgroup (BPP = 100%; BS<50%) consisted of six monophyletic lineages: *T. geminata*, *Theba* sp. 4, *T.* cf. *arinagae*, *T. grasseti*, *Theba* sp. 1a and *Theba* sp. 1b. Population FU3 was split with one individual belonging to *Theba* sp. 1a and the other to *Theba* sp. 1b. Phylogenetic relationships in this subgroup were only weakly supported.

The AFLP data set consisted of 625 specimens, each scored for 1964 loci. Though the AFLP topology was different from that of COI, it supported nearly the same MOTUs (Fig. 3). *T. impugnata* and *Theba* sp. 2 formed a monophyletic group (BS = 100%) and were sister to all other MOTUs (BS = 100%), as in the COI tree. In the cluster comprising all remaining MOTUs (BS = 90%), most splits were only weakly supported with BS values <50%. This cluster comprised *T. grasseti*, *T. geminata*, *Theba* sp.1a, *Theba* sp. 1b, *Theba* sp. 4, *T.* cf. *arinagae*, *Theba* sp. 5 “Sand” and *Theba* cf. *clausoinflata* “Rock”. In the AFLP tree, specimens of FU3 were monophyletic and neither belonged to *Theba* sp. 1a nor to *Theba* sp. 1b. In contrast to COI, *Theba* sp.1b was paraphyletic as specimens of FU7 formed an independent monophyletic lineage in the AFLP phylogeny. In the Jandía group, *Theba* cf. *clausoinflata* “Rock” was not monophyletic. FU13, FU18, FU23, and FU25 formed a separate lineage and were closely related to *Theba* sp. 5 “Sand”. Moreover, FU17 was placed within *Theba* sp. 5 “Sand” and did not belong to *Theba* cf. *clausoinflata* “Rock” as suggested by COI. Most of the basal nodes within the Jandía subgroup had a BS value <50%.

### MOTU assignment

The MOTU assignment test yielded 16 clusters, of which seven (*T. grasseti*, *T. geminata*, *Theba* sp.1a, *Theba* sp. 1b, *T.* cf. *arinagae*, *Theba* sp. 5 “Sand” and *Theba* cf. *clausoinflata* “Rock”) fully agreed with the *a priori* identified MOTUs of the phylogenetic reconstruction of the COI sequences (Fig. 2). According to the assignment test, *Theba* sp. 2 split into four clusters, but none of the four clusters corresponded to any monophyletic subgroup of *Theba* sp. 2 suggested by the COI or AFLP phylogeny (Figs. 2, 3).Therefore we conservatively treated *Theba* sp. 2 as one single MOTU. Moreover, *T. impugnata* splits into three and *Theba* sp. 4 into two clusters. As in both cases additional clusters only contained one specimen each, we refrained from over splitting *T. impugnata* and *Theba* sp. 4 into several MOTUs.

### Morphometrics

**Figure 4:**
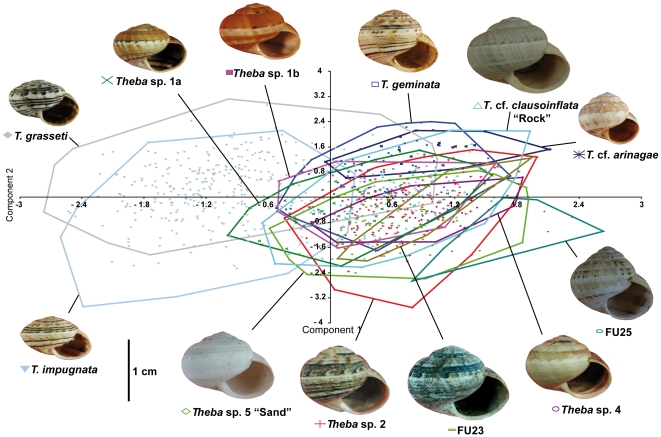
Principal component scatterplot (component 1 versus 2). The analysis was based on geometric morphometrics of ten shell parameters. Convex hulls circumscribe the areas occupied by each MOTU.

In plots following a PCA of the ten landmarks with four significant principal components explaining 87.65% of the total variance, only the flat shelled but very variable *T. grasseti* and *T. impugnata* – both overlapping extensively –, as well as FU25 could be partially separated. The remaining globular-shelled MOTUs overlapped considerably (Fig. 4). The results of a canonical variates analysis (CVA) were similar (not shown). However, the associated MANOVA was highly significant (Wilk's lambda = 0.0183; df = 176, 8545; p<0.0001) and in pairwise Hotelling's comparisons (with four constraints) all MOTUs could be distinguished from each other (p<0.0001 in all cases). FU23 and FU25 had to be compared using Goodall's F-test due to the smaller sample sizes (F = 7.52; df = 16, 288; p<0.0001).

The F-test comparing size across all MOTUs was highly significant (F = 216; df = 131, 4; p<0.0001). We preferred the F-test over an ANOVA due to unequal variances. In Tukey's pairwise post-hoc tests, only *T*. cf. *arinagae* and FU23 as the smallest and largest shelled samples, respectively, could be distinguished from all other MOTUs. *T. geminata* and *Theba* sp. 2 as well as *Theba* sp. 1b and *Theba* sp. 4 could not be distinguished, both pairs exhibiting parapatric distributions. Similarly, *Theba* sp. 5 “Sand”, *Theba* cf. *clausoinflata* “Rock” and FU25 from the Jandía peninsula were not differentiated by size. In contrast, *Theba* sp. 1b was significantly larger than its parapatric sister MOTU, *Theba* sp. 1a (Q = 7.915; p<0.0001) (Fig. S1).

The assignment success based on shell shape ranged from 41.0% (*Theba* sp. 1a) to 95.2% (*T*. cf. *arinagae*). However, the assignments were unambiguous and statistically significant in only 141 of the 961 (14.7%) cases, reflecting the high degree of morphological similarity among the MOTUs (Table S3).

Among mixed samples, eight individuals of LZ23 were measured and investigated genetically. Genetically, four snails belonged to *Theba* sp. 2 and four to *Theba* sp. 1a. Morphologically, the former were all assigned to *Theba* sp. 2, except one. Of the latter, only two were correctly allocated. This indicates that even on a small scale, assignment was ambiguous due to the very similar morphologies of the MOTUs involved. LZ12 and LZ21 were samples consisting of *T. geminata* and *Theba* sp. 2. In LZ12, 12 shells were identified as *T. geminata* and 18 as *Theba* sp. 2. In LZ21, the proportion was 21∶8. In FU3, 13 shells were assigned to *Theba* sp. 1a and ten to *Theba* sp. 1b. As many specimens in these larger samples were empty shells, these samples were excluded from general analyses due the problems with molecular identification and morphological assignment. According to COI, FU17 belonged to MOTU *Theba* cf. *clausoinflata* “Rock”. In contrast, AFLPs placed this sample in MOTU *Theba* sp. 5 “Sand”. Morphological assignment was again ambiguous with two shells identified as “Rock” and three as “Sand”. This sample also was not included in the general analyses.

### Population structure based on the AFLP data set

**Figure 5:**
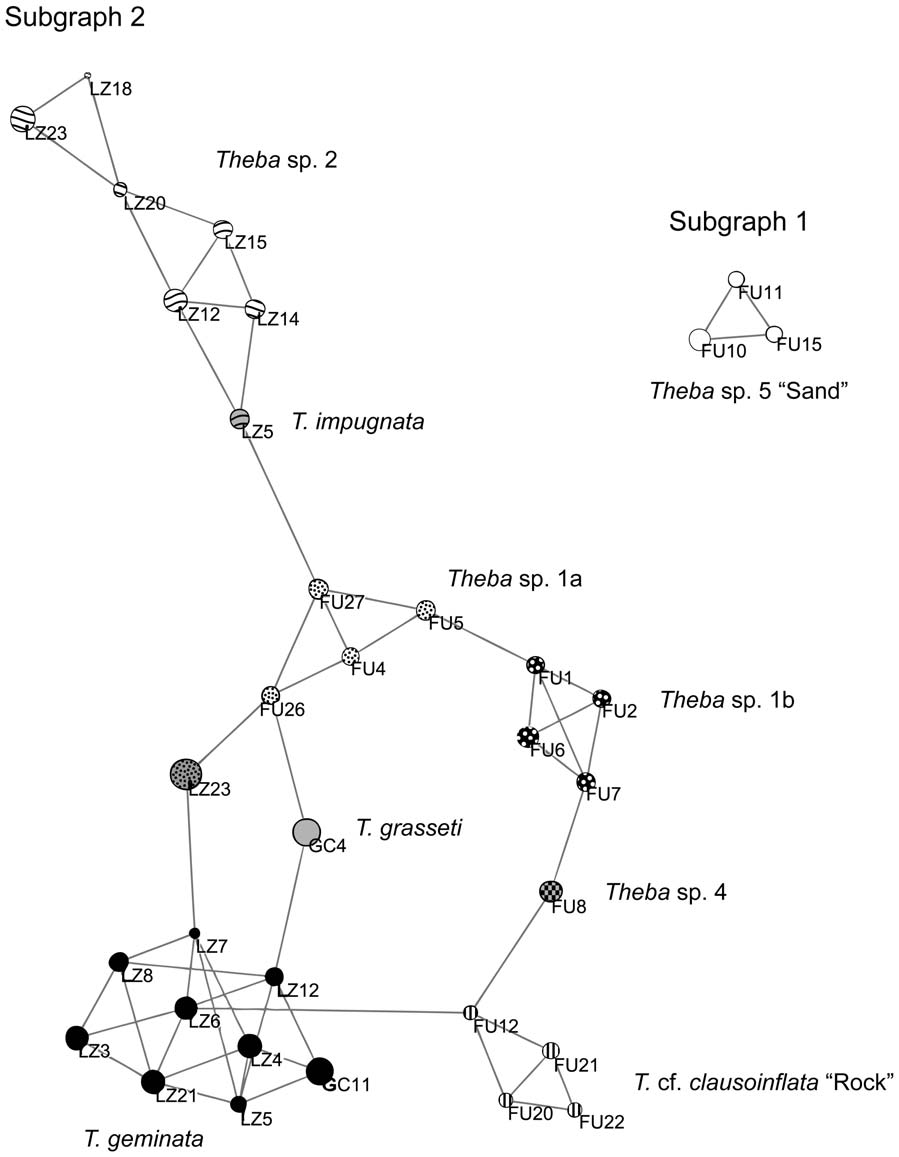
Population graph based on the AFLP data set. The graph represents the genetic covariance relationships among *Theba* populations of the Canary Islands. Node sizes are proportional to within population genetic variability, whereas the edge lengths represent the among population component of genetic variation.

The Population graph of 34 *Theba* populations from Lanzarote, Fuerteventura and Gran Canaria had 56 edges. It consisted of two non-connected subgraphs (Fig. 5), indicating great genetic differentiation between populations of *Theba* sp. 5 “Sand” (subgraph 1) and all remaining populations (subgraph 2).

**Figure 6:**
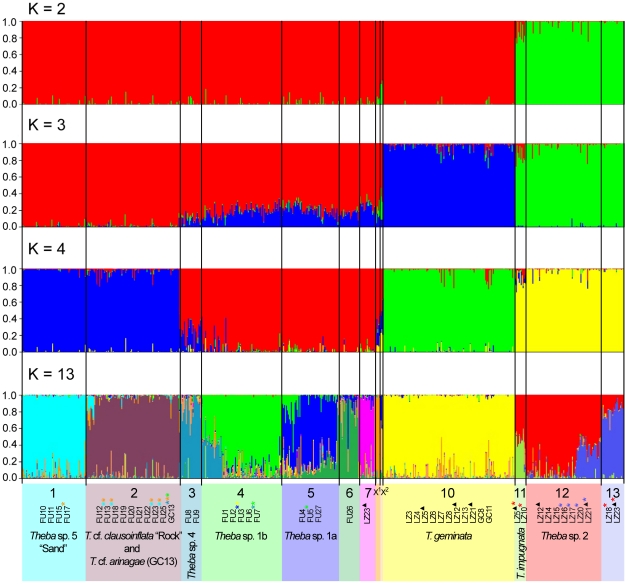
Results of the *Structure* analyses based on the AFLP data set. *Structure* graphical outputs for *K* = 2, *K* = 3, *K* = 4, and *K* = 13 are shown. Each individual is represented by a vertical bar colored in proportion to their estimated ancestry within each cluster. X^1^ = Cluster 8 comprising all individuals of *T. grasseti* (GC3, GC4) and one individual of *T. macandrewiana* (SEL1); X^2^ = Cluster 9 comprising one individual of *T. macandrewiana* (SEL1) and two individuals of FU4; * = highly admixed populations, which also showed proportions of the cluster of the corresponding color; ▴ = mixed sample (i.e. samples are composed of two species).

Results of the Bayesian clustering analyses using *Structure* v2.3.2 were largely congruent with those of previous molecular analyses of the present study. The mean likelihood *L*(*K*) increased from *K* = 1 to a maximum value at *K* = 13 (−340259.27) and then decreased to a minimum value at *K* = 45 (−4266627.79), whereas the variance of *L*(*K*) between runs increased for larger *K*s (Fig. S2). The statistic Δ*K* described by Evanno et al. [53] showed multiple peaks at *K* = 2 (Δ*K* = 935.36), *K* = 3 (Δ*K* = 550.49) and *K* = 4 (Δ*K* = 194.65). The highest mean likelihood value of *L*(*K*) at *K* = 13 was confirmed by a fourth peak of Δ*K* = 20.26 (Fig. S2). The graphical outputs of *Structure* (from the run at each *K* with the highest likelihood) for *K* = 2, 3, 4 and 13 are shown in Fig. 6. At *K* = 2, individuals of *Theba* sp. 2 and *T. impugnata* grouped into one cluster and all remaining individuals into the other cluster. At *K* = 3, individuals of *T. geminata* were assigned to a separate third cluster. At *K* = 4, a fourth cluster comprised all individuals sampled on the Jandía peninsula. In the run that had the highest likelihood at *K* = 13, nine clusters corresponded to MOTUs identified by the prior molecular analyses (above): *Theba* sp. 5 “Sand”, *T.* cf. *clausoinflata* “Rock”, *Theba* sp. 4, *Theba* sp. 1a, *Theba* sp. 1b, *T. grasseti*, *T. geminata*, *T. impugnata* and *Theba* sp. 2. Individuals of LZ18 and of *Theba* sp. 2 of LZ23 were assigned to an additional cluster, as were individuals of *Theba* sp. 1a of LZ23. Furthermore, individuals of FU26 grouped into a separate cluster. Another cluster did not correspond to any population or MOTU and comprised two individuals of FU4 and one specimen of *T. macandrewiana*

At *K* = 13, most individuals were assigned to one cluster with 70% to 99% probability. Nevertheless, some individuals showed high levels of admixture and had proportions much lower than this. Individuals of the cluster *T. impugnata* were highly admixed and showed proportions of up to 62% of the cluster *Theba* sp. 2. *Theba* sp. 2 included populations (LZ16, LZ17, LZ21 and LZ20) in which individuals had levels of admixture >49% with cluster LZ18 & LZ23. The assignment of FU7 and FU5 to a single cluster was not obvious. FU7 showed mixed ancestry with proportions of the clusters *Theba* sp. 4 (24–48%) and *Theba* sp. 1b (24–75%). Though individuals of the population FU5 were mainly assigned to the cluster *Theba* sp. 1a, some individuals showed levels of admixture of up to 68% of the cluster *Theba* sp. 1b. Mainly assigned to *Theba* sp. 5 “Sand”, FU17 were admixed and showed proportions of the cluster *T. grasseti*. Corresponding to the COI phylogeny, FU25, FU23, FU18 and FU13 were associated with *T.* cf. *clausoinflata* “Rock”, but additionally had proportions of the clusters *Theba* sp. 5 “Sand” and *T. grasseti*. Both specimens of FU3 grouped into *Theba* sp. 1b, although one individual was admixed and showed proportions of the clusters *Theba* sp. 1a and *T. geminata*. Neither specimens of *T.* cf. *arinagae* nor those of *T. macandrewiana* were assigned to a discrete genetic cluster. *T.* cf. *arinagae* grouped with highest proportion into *T.* cf. *clausoinflata* “Rock”, but it was highly admixed and showed proportions of the clusters *Theba* sp. 4, *T. grasseti* and *Theba* sp. 1b. *T. macandrewiana* was split with one individual assigned to the cluster *T. grasseti* and the other to the cluster, which did not correspond to any population or MOTU (cluster 9, see Fig. 6).

A non-hierarchical AMOVA of the whole data set indicated great population differentiation (F_ST_ = 0.24) [62] (Table S2). Separate non-hierarchical analyses of Lanzarote and Fuerteventura, respectively, showed that genetic variation among Lanzarote populations (27%) was higher than that among Fuerteventura populations (16%). A nested AMOVA of Lanzarote populations indicated substantial genetic differentiation between populations of *T. geminata* and populations of *T. impugnata* and *Theba* sp. 2 (F_CT_ = 0.28). On Fuerteventura, a hierarchical AMOVA showed the highest among group variation between populations of *T.* cf. *clausoinflata* “Rock” and *Theba* sp. 5 “Sand” (14.8%). Whereas genetic differentiation between populations of *Theba* sp. 5 “Sand” was moderate (F_ST_ = 0.05), it was low between populations of *T.* cf. *clausoinflata* “Rock” (F_ST_ = 0.04). A hierarchical AMOVA indicated moderate genetic differentiation between Jandía populations and populations of the main part of Fuerteventura (F_CT_ = 0.08). Genetic divergence between populations of *Theba* sp. 1a and *Theba* sp. 1b was low (F_CT_ = 0.04). A hierarchical AMOVA between Lanzarote and Fuerteventura populations revealed that only 8.4% of the variation was explained by between-group differences. The variation among groups increased to 21.3%, by associating *T. geminata* from Lanzarote with populations of Fuerteventura. The AMOVA, structured according to the MOTUs suggested by the phylogenetic analyses and the MOTU assignment test, attributed 19.2% of the global variation to differences among MOTUs, and 7.1% to differentiation among populations within MOTUs. All fixation indices were significant. Conversely, hierarchical AMOVA among populations of *Theba* sp. 2 and *T. impugnata* revealed no significant genetic differentiation. In the light of the results of the Population graphs and *Structure* analyses, we further investigated genetic structure within *Theba* sp. 1a. LZ23 of Lanzarote and FU26 of the Isle of Lobos (see Fig. 1) were each compared with the other populations of *Theba* sp. 1a. The genetic differentiation among populations of *Theba* sp. 1a was also non-significant.

### Isolation by distance

**Figure 7:**
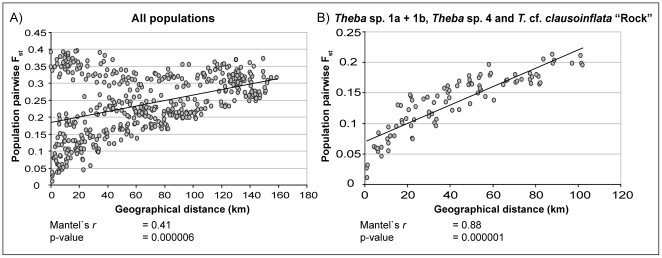
Isolation by distance (IBD). A) Pairwise F_ST_ values for all populations based on AFLP data versus geographical distances between all sample sites in kilometers. B) Pairwise F_ST_ values for all populations of *Theba* sp. 1a, *Theba* sp. 1b, *Theba* sp. 4 and *T.* cf.*clausoinflata* “Rock” based on AFLP data versus geographical distances between corresponding sample sites in kilometers.

A Mantel test including all populations of Lanzarote and Fuerteventura indicated evidence for IBD (Pearson correlation coefficient r = 0.41, p<0.001) (Fig. 7; Table S4). The correlation between genetic and geographic distance increased, however, by successively excluding populations of a) *T. impugnata* and *Theba* sp. 2 (r = 0.82, p<0.001) and b) *Theba* sp. 5 “Sand” (r = 0.84, p<0.001) from the data set. Finally removing *T. geminata* induced the highest correlation value (r = 0.88, p<0.001) and indicated strong patterns of IBD among populations of *Theba* sp. 1a, *Theba* sp. 1b, *Theba* sp. 4 and *T.* cf. *clausoinflata* “Rock” on Fuerteventura. A separate Mantel test of all populations from Lanzarote yielded the lowest correlation value (r = 0.27, p<0.001). Excluding *T. geminata* from this data set, increased the correlation value to r = 0.75 (p<0.001).

### Outlier detection

BayeScan detected outlier loci in almost every population comparison. The average number of outlier loci was highest in MOTU pairs of Fuerteventura and lowest in those of Lanzarote (Table 1). Consistent outlier loci were found in six MOTU pairs. *Theba* sp. 4 and *T. impugnata*, however, were each represented by only one population. Thus, consistent outlier loci detected in the three MOTU pairs *Theba* sp. 4/*Theba* sp. 1b, *T. impugnata*/*Theba* sp. 4, and *T. geminata*/*Theba* sp. 4 were not further considered, as they were potentially linked to population-specific demographic history, unrelated to ecological pressure. BayeScan identified three consistent outlier loci (1199, 1488, and 1801) between populations of *Theba* sp. 5 “Sand” and populations of *T.* cf. *clausoinflata* “Rock”, indicating genotype-environment associations (GEAs) on the Jandía peninsula. Population comparisons within MOTU pair *Theba* sp. 5 “Sand”/*Theba* sp. 1b revealed one (1488) and within MOTU pair *Theba* sp. 5 “Sand”/*Theba* sp. 4 three consistent outlier loci (788, 1488 and 1801). Though MOTU pair *Theba* sp. 5 “Sand”/*Theba* sp. 4 involved always the same population of *Theba* sp. 4 in each comparison (as explained above), two of the three consistent outlier loci were 1488 and 1801. These loci were apparently linked to populations of *Theba* sp. 5 “Sand”.

**Table 1:** Outlier loci detection.

Island	MOTU pairs			Average # of outlier loci	Consistent outlier loci
**Jandía,**	*Theba* sp. 5 “Sand” (3)	vs.	*T.* cf. *clausoinflata* “Rock” (4)	7.92	1199, 1488, 1801
**Fuerteventura**	*Theba* sp. 5 “Sand” (3)	vs.	*Theba* sp. 5 “Sand” (3)	1.00	–
	*T.* cf. *clausoinflata* “Rock” (4)	vs.	*T.* cf. *clausoinflata* “Rock” (4)	1.00	–
**Fuerteventura**	*Theba* sp. 5 “Sand” (3)	vs.	*Theba* sp. 4 (1)	10.33	788, 1488, 1801
	*Theba* sp. 5 “Sand” (3)	vs.	*Theba* sp. 1a (5)	4.07	–
	*Theba* sp. 5 “Sand” (3)	vs.	*Theba* sp. 1b (4)	9.67	1488
	*T.* cf. *clausoinflata* “Rock” (4)	vs.	*Theba* sp. 4 (1)	2.75	–
	*T.* cf. *clausoinflata* “Rock” (4)	vs.	*Theba* sp. 1a (5)	2.10	–
	*T.* cf. *clausoinflata* “Rock” (4)	vs.	*Theba* sp. 1b (4)	3.81	–
	*Theba* sp. 1a (5)	vs.	*Theba* sp. 1b (4)	1.25	–
	*Theba* sp. 4 (1)	vs.	*Theba* sp. 1a (5)	2.60	–
	*Theba* sp. 4 (1)	vs.	*Theba* sp. 1b (4)	7.25	1064, 1068, 1187
	*Theba* sp. 1a (5)	vs.	*Theba* sp. 1a (5)	0.90	–
	*Theba* sp. 1b (4)	vs.	*Theba* sp. 1b (4)	1.83	–
**Lanzarote**	*Theba geminata* (8)	vs.	*Theba* sp. 2 (5)	0.80	–
	*Theba geminata* (8)	vs.	*Theba impugnata* (1)	0.88	–
	*Theba* sp. 2 (5)	vs.	*Theba impugnata* (1)	1.00	–
	*Theba* sp. 2 (5)	vs.	*Theba* sp. 2 (5)	1.00	–
	*Theba geminata* (8)	vs.	*Theba geminata* (8)	1.00	–
**Fuerteventura**	*Theba* sp. 2 (4)*	vs.	*Theba* sp. 1a (5)	0.95	–
**Lanzarote**	*Theba* sp. 2 (4)*	vs.	*Theba* sp. 1b (4)	1.00	–
	*Theba* sp. 2 (4)*	vs.	*Theba* sp. 4 (1)	1.75	–
	*Theba impugnata* (1)	vs.	*Theba* sp. 1a (5)	1.00	–
	*Theba impugnata* (1)	vs.	*Theba* sp. 1b (4)	0.75	–
	*Theba impugnata* (1)	vs.	*Theba* sp. 4 (1)	1.00	1064
	*Theba geminata* (2)*	vs.	*Theba* sp. 1a (5)	0.90	–
	*Theba geminata* (2)*	vs.	*Theba* sp. 1b (4)	1.38	–
	*Theba geminata* (2)*	vs.	*Theba* sp. 4 (1)	1.00	1064
	*Theba geminata* (2)*	vs.	*T.* cf. *clausoinflata* “Rock” (4)	0.86	–
	*Theba geminata* (2)*	vs.	*Theba* sp. 5 “Sand” (3)	0.83	–

Analyses were based on 1964 AFLP markers using BayeScan. It estimates a posterior probability for each locus being under selection. All loci with a posterior probability over 95% were retained as outliers. For each MOTU pair, all possible pairwise comparisons between populations were tested.

() values in brackets indicate the number of populations of each MOTU.

* only a subset of populations of the corresponding MOTU was analyzed.

## Discussion

### Cryptic diversification

Currently, only two extant species of *Theba* are described from Fuerteventura and Lanzarote - the flat-shelled *T. impugnata* and the globular-shelled *T. geminata* [15]–[17]. In the present study, *Theba* populations on Lanzarote and Fuerteventura displayed little divergence in shell morphology. Apart from the flat-shelled *T. grasseti* from Gran Canaria, a PCA based on ten shell parameters largely separated only the flat-shelled *T. impugnata* (Lanzarote) and snails from the Pico de la Zarza (FU25) from all remaining globular-shelled snails (Fig. 4). Molecular data from these *Theba* populations, however, showed extensive genetic differentiation among globular-shelled snails of *Theba* on both islands. Aside from the flat-shelled *T. impugnata*, molecular results supported two endemic globular-shelled MOTUs on Lanzarote and five on Fuerteventura (Figs. 2, 3; Table S2). Morphologically clearly distinct snails from the Pico de la Zarza (FU25), however, were not genetically differentiated from the globular-shelled group. Only in the case of *T. impugnata*, did the distinct shell shape correspond to its high genetic differentiation. In summary, we observed extensive cryptic diversification among globular-shelled *Theba* populations on both islands.

### Allopatric divergence

*T. impugnata* is restricted to the north of Lanzarote, whereas *Theba* sp. 2 is found in the western parts of central and south Lanzarote (Fig. 1; Table S1). Until now both forms have not been found in sympatry. The AMOVA revealed that genetic differentiation between *T. impugnata* and *Theba* sp. 2 was not significant (Table S2). *T. impugnata*, however, is morphologically clearly differentiated from *Theba* sp. 2 (Fig. 4) and *Structure* analyses demonstrated allele frequency divergence between both MOTUs (Fig. 6). Within the large range of *Theba* sp. 2, we observed a clear differentiation between the most southern (LZ18 and LZ23) and northern populations (LZ14 and LZ15). The populations in between (LZ16, LZ17, LZ21 and LZ20) displayed mixed ancestry suggesting gene flow among adjacent populations due to the lack of reproductive isolation (Figs. 1 and 6). The discontinuous distribution of genetic divergence and mixture is compatible with documented historical (<500 years) volcanic activity on Lanzarote [12] possibly eradicating snails in the central area of the island, followed by allopatric differentiation of the most southern and northern populations and secondary contact.

*T. geminata* is distributed in north and central Lanzarote, and its southernmost populations occur sympatrically with either *T. impugnata* or *Theba* sp. 2. There was no evidence of gene flow (Fig. 6), suggesting that *T. geminata* is reproductively isolated from both *T. impugnata* and *Theba* sp. 2. Due to the Pleistocene's low sea-level, Lanzarote and Fuerteventura were periodically connected by land bridges, providing enhanced dispersal opportunities between them [63]–[64]. We propose that Fuerteventura was colonized from Lanzarote, with *T. geminata* closely related to MOTUs of Fuerteventura (Figs. 2, 3 and 5; Table S2). This interpretation, however, is tentative as phylogenetic relationships were not well supported.

We found a clear pattern of isolation by distance (IBD) among populations on Fuerteventura (Fig. 7; Table S4). *Structure* analyses revealed that differentiation processes on the main part of Fuerteventura were similar to those of *Theba* sp. 2 on Lanzarote. Excluding populations with highly admixed proportions (FU3, FU5 and FU7) (Fig. 6), the ranges of *Theba* sp. 4, *Theba* sp. 1b and *Theba* sp. 1a are separated by mountain ranges possibly resulting from the volcanic history of Fuerteventura [12], [65]. In contact zones (FU3, FU5 and FU7), however, the *Structure* analyses suggested gene flow among adjacent populations. As with *Theba* sp. 2 on Lanzarote, this pattern of genetic differentiation is compatible with allopatric divergence of *Theba* sp. 4, *Theba* sp. 1b and *Theba* sp. 1a, followed by hybridization on secondary contact due to a lack of reproductive isolation.

Jandía populations were distinct from populations on the main part of the island (Figs. 2, 3 and 6; Table S2), indicating that the Isthmus of La Pared is probably a barrier to gene flow. The sand dunes at the Isthmus of La Pared are a barrier to gene flow in other organisms [66], which apparently leads to allopatric divergence of species between the southern peninsula and the main part of the island.

In all cases the BayeScan analyses did not found any consistent outlier loci, suggesting that differentiation processes of *Theba* on Fuerteventura and Lanzarote were probably driven mainly by non-adaptive allopatric differentiation.

### Ecological differentiation

Speciation in non-adaptive radiations is often slow. It depends on the accidental occurrence and fixation of different alleles in allopatric populations in ecologically similar environments. Many allopatric populations hybridize when they meet in secondary contact zones [67]–[69]. In contrast, ecological speciation can be rapid, developing reproductive isolation within a few thousands of years [70]–[71].

In contrast to the main part of Fuerteventura, the Jandía peninsula is characterized by high mountains and great habitat diversity [11]. *Theba* sp. 5 “Sand” and *T.* cf. *clausoinflata* “Rock” are parapatrically distributed on the Jandía peninsula without any obvious geographical barriers. Both formed stable genetic clusters with great genetic differentiation from all other Fuerteventuran MOTUs (Figs. 2, 3 and 6; Table S2). Whereas *Theba* sp. 5 “Sand” is restricted to sandy environments, *T.* cf. *clausoinflata* “Rock” is exclusively found in rocky habitats. BayeScan analyses helped to identify genetic loci that are more divergent between *Theba* sp. 5 “Sand” and *T.* cf. *clausoinflata* “Rock” than expected under neutrality, indicating genotype-environment associations (GEAs) and possibly ecologically driven differentiation (Table 1). Since both MOTUs are endemic to Jandía, parapatrically distributed, sister groups, and genetically differentiated, a sympatric/parapatric speciation scenario involving divergent selection within ecologically different environments seems plausible [18]–[19], [72]–[73]. This was also supported by the Population graph indicating greatest genetic differentiation between *Theba* sp. 5 “Sand” and all other MOTUs from Lanzarote and Fuerteventura (Fig. 5) mainly restricted to compact-soil and rocky habitats.

Bierne et al. [74] argued, however, that endogenous (i.e. environment-independent) genetic incompatibilities coinciding with environmental boundaries rather than local adaptation are often more likely to explain GEAs. The occurrence of outlier loci would thus be an accidental by-product of demographic history and present genetic isolation. This hypothesis would predict that multiple independent comparisons across different ecological environments would result in randomly distributed outlier loci within genomes. As populations of *Theba* sp. 5 “Sand” are discontinuously distributed on Jandía and multiple comparisons between “Sand” and “Rock” populations revealed a common set of outlier loci, we considered this set as GEAs best explained by ecological adaptation (Table 1). The presence of GEAs and strong genetic differentiation, however, are not sufficient indications of a sympatric/parapatric speciation scenario. Microallopatric speciation might also play an important role in organisms with low dispersal abilities like land snails [75]–[77]. Further evidence is needed to test if *Theba* sp. 5 “Sand” and *T.* cf. *clausoinflata* “Rock” actually diverged in allopatry, parapatry or sympatry.

Though populations found on the top of Pico de la Zarza (FU25) and the upper parts of Degollada de Vinamar (FU23) as well as in rocky areas near the coast (FU13 and FU18) were associated with *T.* cf. *clausoinflata* “Rock”, they were admixed with proportions of *Theba* sp. 5 “Sand” (Figs. 1 and 6). However, as these populations were each represented by only two individuals, we refrain from speculation on the reasons for this.

## Supporting Information

Dataset S1 **AFLP binary character matrix including replicates.**
(TXT)

Figure S1 **Box plots of centroid size for each** ***Theba*** **MOTU.** The horizontal line represents the mean, box margins are at the 25th and 75th percentiles, and bars extend to the 5th and 95th percentiles.
(DOC)

Figure S2 **Estimation criteria for the number of genetic clusters in the AFLP data set.** (A) *K* vs. mean *L(K)* with standard deviation (SD) from 10 replicates for each K. (B) *K* vs. Δ*K* (following Evanno et al. [53]).
(DOC)

Table S1 **Summary of specimens used in the present study.**
(DOC)

Table S2 **Summary of AMOVA with and without different hierarchies.** Populations with fewer than 5 individuals were excluded from analysis due to small sample size (see Table S1). MOTUs corresponded to results of phylogenetic analyses and the MOTU assignment test. Bold fixation indices were statistically significant.
(DOC)

Table S3 **Canonical variates analysis (CVA) based assignment success.** *Theba* MOTUs along rows, CVA groups along columns.
(DOC)

Table S4 **Results of the simple Mantel test analyses.** In the analyses matrices of geographical distance (km) were compared to genetic distances (F_ST_).
(DOC)
