# Correction: Radiating on Oceanic Islands: Patterns and Processes of Speciation in the Land Snail Genus *Theba* (Risso 1826)

**DOI:** 10.1371/annotation/5cfe7c14-9385-427b-ac34-7e893af0cb8c

**Published:** 2012-09-10

**Authors:** Carola Greve, France Gimnich, Rainer Hutterer, Bernhard Misof, Martin Haase

There were errors in the Author Contributions section. The correct contributions are: Conceived and designed the experiments: RH MH BM CG. Performed the experiments: CG FG MH. Analyzed the data: CG FG MH. Wrote the paper: CG BM MH. Carried out the field work: CG FG RH.

Also, there was an error in reference 46. The correct reference 46 information should read: 46. Haase M, Misof B (2009) Dynamic gastropods: stable shell polymorphism despite gene flow in the land snail Arianta arbustorum. J Zool Syst Evol Res 47:105-114. 

