# Supplementary material for: Radiating on Oceanic Islands: Patterns and Processes of Speciation in the Land Snail Genus Theba (Risso 1826)
Source: PLoS One. 2012 Apr 6;7(4):e34339. doi: 10.1371/journal.pone.0034339 (PMC3321021; doi:10.1371/journal.pone.0034339)
Supplement: Figure S1 — Box plots of centroid size for each Theba MOTU. The horizontal line represents the mean, box margins are at the 25th and 75th percentiles, and bars extend to the 5th and 95th percentiles. (DOC) [file pone.0034339.s002.doc]

**
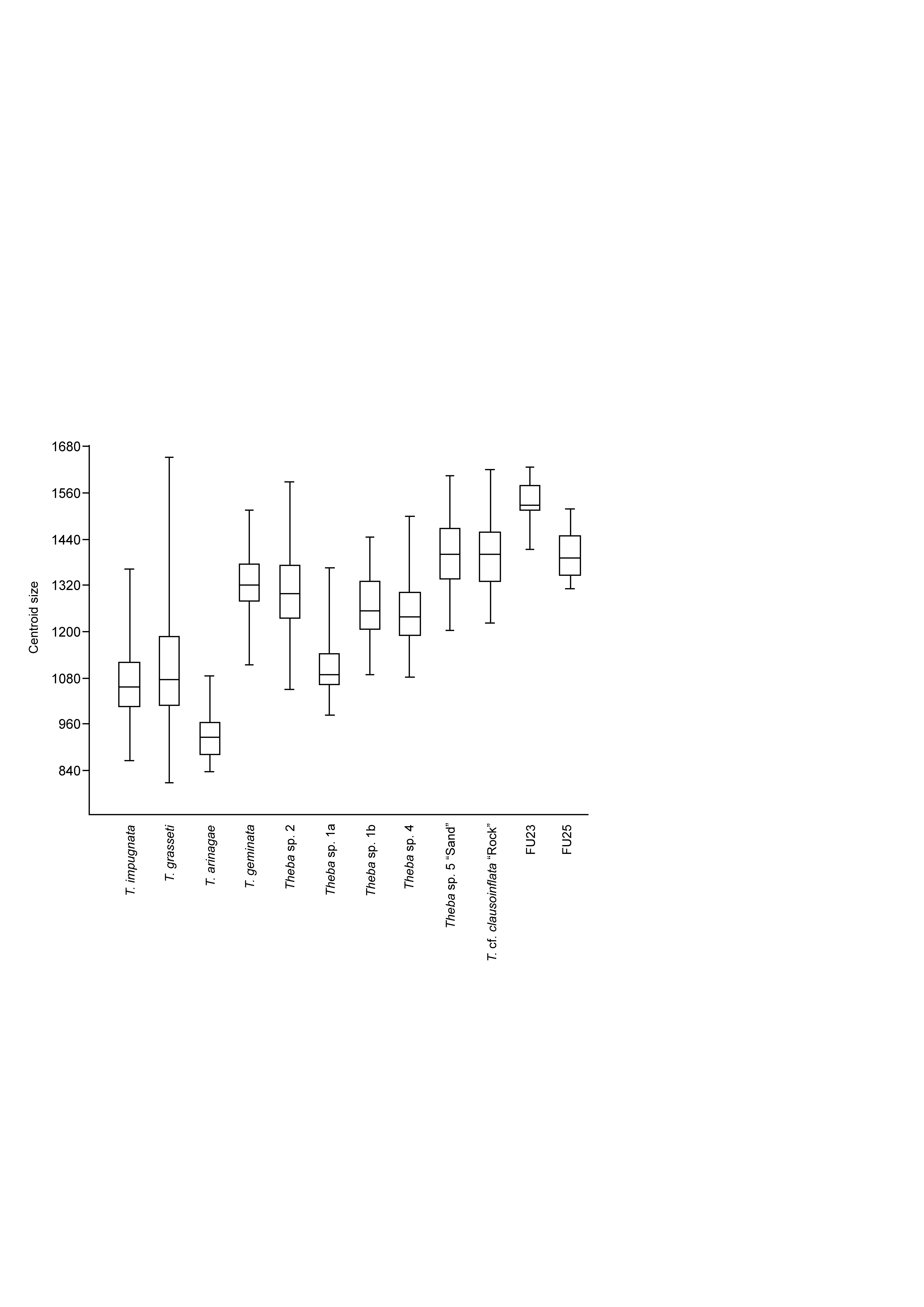
**

**Figure S1.** Box plots of centroid size for each *Theba* MOTU. The horizontal line represents the mean, box margins are at the 25th and 75th percentiles, and bars extend to the 5th and 95th percentiles.
