# Supplementary material for: Radiating on Oceanic Islands: Patterns and Processes of Speciation in the Land Snail Genus Theba (Risso 1826)
Source: PLoS One. 2012 Apr 6;7(4):e34339. doi: 10.1371/journal.pone.0034339 (PMC3321021; doi:10.1371/journal.pone.0034339)
Supplement: Figure S2 — Estimation criteria for the number of genetic clusters in the AFLP data set. (A) K vs. mean L(K) with standard deviation (SD) from 10 replicates for each K. (B) K vs. ΔK (following Evanno et al. [53]). (DOC) [file pone.0034339.s003.doc]

**
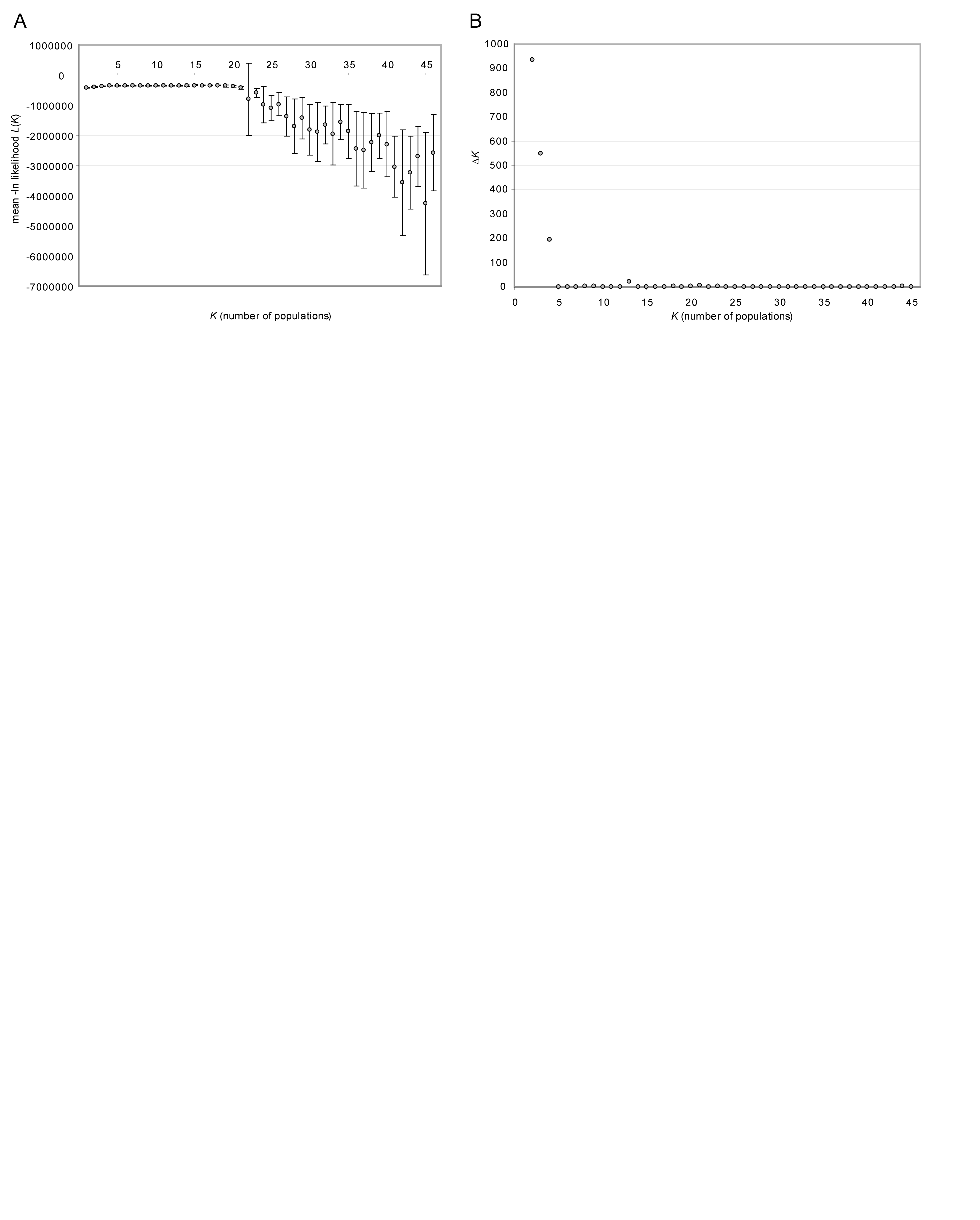
**

**
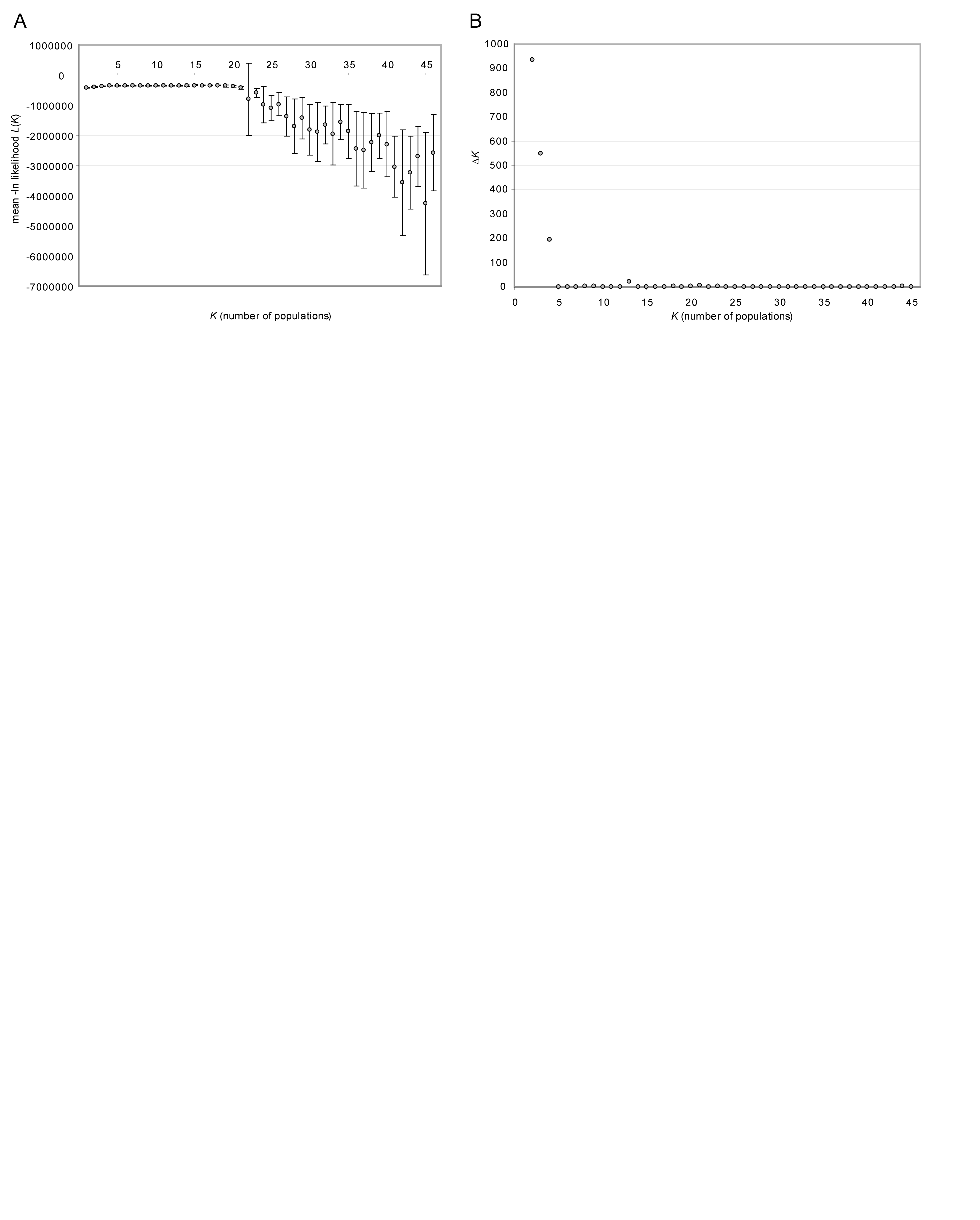
**

**Figure S2.** Estimation criteria for the number of genetic clusters in the AFLP data set. (A) *K* vs. mean *L(K)* with standard deviation (SD) from 10 replicates for each K. (B) *K* vs. Δ*K* (following Evanno et al. [53]).
