# Supplementary material for: Radiating on Oceanic Islands: Patterns and Processes of Speciation in the Land Snail Genus Theba (Risso 1826)
Source: PLoS One. 2012 Apr 6;7(4):e34339. doi: 10.1371/journal.pone.0034339 (PMC3321021; doi:10.1371/journal.pone.0034339)
Supplement: Table S1 — Summary of specimens used in the present study. (DOC) [file pone.0034339.s004.doc]

**Table S1.** Summary of specimens used in the present study.

| **Species** | **Code** | **Locality ^(a)^** | **Latitude** | **Longitude** | **# of specimens genotyped (AFLP)** | **# of specimens used in morphometrics** | **Collec-tion ^(b)^** | **COI GenBank Accession #** |
| --- | --- | --- | --- | --- | --- | --- | --- | --- |
| *Theba geminata* | LZ3 | LZ, between Arrieta and Haría | N29°08.481’ | W13°28.922’ | 22 | 25 | ZFMK | JN408144-408145 |
| *Theba geminata* | LZ4 | LZ, Mirador los Helechos | N29°07.672’ | W13°30.847’ | 14 | ­- | ZFMK | JN408146-408147 |
| *Theba geminata* | LZ5* | LZ, road to Mirador de Haría | N29°07.893’ | W13°30.912’ | 18 | ­- | ZFMK | JN408148-408150 |
| *Theba geminata* | LZ6 | LZ, between Maguez and Haría | N29°09.082’ | W13°30.022’ | 20 | ­- | ZFMK | JN408151-408153 |
| *Theba geminata* | LZ7 | LZ, Mirador de Guinate | N29°11.099’ | W13°30.064’ | 8 | 25 | ZFMK | JN408154-408155 |
| *Theba geminata* | LZ8 | LZ, Mirador del Rio | N29°12.799’ | W13°28.869’ | 19 | 24 | ZFMK | JN408156-408158 |
| *Theba geminata* | LZ12* | LZ, S Teguise | N29°02.981’ | W13°33.793’ | 10 | 12 | ZFMK | JN408159-408160 |
| *Theba geminata* | LZ21* | LZ, 1.5 km SW San Bartolomé | N28°59.431’ | W13°37.352’ | 20 | 21 | ZFMK | JN408180, JN408182 |
| *Theba geminata* | GC8/11 | GC, Anden Verde | N28°01.992’ | W15°45.432’ | 4 | ­ | ZFMK | HM034468, JN408136-408138 |
| *Theba impugnata* | LZ5* | LZ, road to Mirador de Haría | N29°07.893’ | W13°30.912’ | 7 | in total 188^■^ | ZFMK | HM114297 - 114303 |
| *Theba impugnata* | LZ10 | LZ, 4 km S Orzola | N29°11.690’ | W13°27.628’ | 2 |  | ZFMK | HM114304 - 114305 |
| *Theba grasseti* | GC3 | GC, Punta de Arena | N28°02.424’ | W15°45.864’ | 2 | in total 175^■^ | ZFMK | HM034542, JN408139 |
| *Theba grasseti* | GC4 | GC, La Isleta | N28°09.552’ | W15°25.992’ | 3 |  | ZFMK | HM034543, JN408142 |
| *Theba* cf. *arinagae* | GC13 | GC, Pico de Arinaga | N27°52.167’ | W15°23.832’ | 1 | 21 | ZFMK | JN408143 |
| *Theba macandrewiana* | SEL1 | P, Selvagem Grande | N30°08.567’ | W15°51.917’ | 2 | ­- | ZFMK | JN408140-408141 |
| *Theba* sp. 2 | LZ12* | LZ, S Teguise | N29°02.981’ | W13°33.793’ | 10 | 18 | ZFMK | JN408161 |
| *Theba* sp. 2 | LZ13 | LZ, way down to Playa del Risco | N29°11.794’ | W13°29.541’ | 2 | ­- | ZFMK | JN408162-408163 |
| *Theba* sp. 2 | LZ14 | LZ, 5 km S Famara | N29°05.156’ | W13°34.230’ | 22 | 30 | ZFMK | JN408164-408166 |
| *Theba* sp. 2 | LZ15 | LZ, 1.5 km NW Sóo | N29°06.249’ | W13°37.880’ | 21 | 28 | ZFMK | JN408167-408169 |
| *Theba* sp. 2 | LZ16 | LZ, La Santa | N29°06.717’ | W13°39.378’ | 2 | 18 | ZFMK | JN408170-408171 |
| *Theba* sp. 2 | LZ17 | LZ, Mácher | N28°56.692’ | W13°42.149’ | 2 | ­- | ZFMK | JN408172-408173 |
| *Theba* sp. 2 | LZ18 | LZ, Valle de Femés | N28°55.113’ | W13°46.506’ | 20 | 30 | ZFMK | JN408174-408176 |
| *Theba* sp. 2 | LZ20 | LZ, Tinajo | N29°03.803’ | W13°40.787’ | 21 | 30 | ZFMK | JN408177-408179 |
| *Theba* sp. 2 | LZ21* | LZ, 1.5 km SW San Bartolomé | N28°59.431’ | W13°37.352’ | 2 | 8 | ZFMK | JN408181 |
| *Theba* sp. 2 | LZ23* | LZ, 2 km N Playa Blanca | N28°52.667’ | W13°49.961’ | 4 | 4 | ZFMK | JN408185, JN408187 |
| *Theba* sp. 1a | LZ23* | LZ, 2 km N Playa Blanca | N28°52.667’ | W13°49.961’ | 17 | 4 | ZFMK | JN408183-408184, JN408186, JN408188-408193 |
| *Theba* sp. 1a | FU4 | FU, 4 km S Corralejo | N28°42.292’ | W13°52.870’ | 20 | 21 | ZFMK | JN408087-408089 |
| *Theba* sp. 1a | FU5 | FU, La Oliva | N28°36.800’ | W13°55.547’ | 21 | 3 | ZFMK | JN408090-408092 |
| *Theba* sp. 1a | FU26 | FU, Lobos, Playa | N28°44.549’ | W13°49.519’ | 22 | 25 | ZFMK | JN408122-408123 |
| *Theba* sp. 1a | FU27 | FU, 1.7 km E Lajares | N28°40.596’ | W13°55.260’ | 19 | 8 | ZFMK | JN408124-408125 |
| *Theba* sp. 1a^♦^ | FU3* | FU, Puerto de Lajas | N28°32.336’ | W13°50.538’ | 1 | 13 | ZFMK | JN408127 |
| *Theba* sp. 1b^♦^ | FU3* | FU, Puerto de Lajas | N28°32.336’ | W13°50.538’ | 1 | 10 | ZFMK | JN408126 |
| *Theba* sp. 1b | FU1 | FU, 5 km SE Triquivijate | N28°24.772’ | W13°55.700’ | 20 | 20 | ZFMK | JN408081-408083 |
| *Theba* sp. 1b | FU2 | FU, Antigua | N28°25.250’ | W14°00.684’ | 19 | 7 | ZFMK | JN408084-408086 |
| *Theba* sp. 1b | FU6 | FU, Tefia | N28°31.478’ | W14°00.068’ | 20 | 6 | ZFMK | JN408093-408095 |
| *Theba* sp. 1b | FU7 | FU, Tuineje | N28°19.761’ | W14°03.207’ | 21 | 20 | ZFMK | JN408096-408098 |
| *Theba* sp. 4 | FU8 | FU, between Pájara and Pared | N28°15.306’ | W14°11.162’ | 22 | 20 | ZFMK | JN408099-408101 |
| *Theba* sp. 4 | FU9 | FU, Pared | N28°12.792’ | W14°13.079’ | 1 | 11 | ZFMK | JN408102-408103 |
| *Theba* sp. 5 "Sand" | FU10 | FU, 2 km NE Costa Calma | N28°10.541’ | W14°12.442’ | 22 | 26 | ZFMK | JN408104-408105 |
| *Theba* sp. 5 "Sand" | FU11 | FU, 5 km W Costa Calma | N28°08.909’ | W14°15.119’ | 21 | 11 | ZFMK | JN408106-408107 |
| *Theba* sp. 5 "Sand" | FU15 | FU, 1 km E Morro de Potala | N28°04.918’ | W14°28.311’ | 22 | 30 | ZFMK | JN408110-408111 |
| *Theba* cf. *clausoinflata* "Rock"*^♦^* | FU17 | FU, Playa de Cofete | N28°06.498’ | W14°23.277’ | 2 | 5 | ZFMK | JN408130-408131 |
| *Theba* cf. *clausoinflata* "Rock" | FU12 | FU, Barranco los Canarios | N28°06.127’ | W14°17.080’ | 21 | 30 | ZFMK | JN408108-408109 |
| *Theba* cf. *clausoinflata* "Rock" | FU13 | FU, 10 km W Morro del Jable | N28°04.256’ | W14°25.286’ | 2 | 30 | ZFMK | JN408128-408129 |
| *Theba* cf. *clausoinflata* "Rock" | FU18 | FU, 11.5 km W Morro del Jable | N28°04.580’ | W14°25.699’ | 2 | 23 | ZFMK | JN408132-408133 |
| *Theba* cf. *clausoinflata* "Rock" | FU19 | FU, Degollada de Vinamar (200 m) | N28°03.983’ | W14°19.824’ | 2 | 29 | ZFMK | JN408134-408135 |
| *Theba* cf. *clausoinflata* "Rock" | FU20 | FU, Degollada de Vinamar (300 m) | N28°04.547’ | W14°20.094’ | 22 | 7 | ZFMK | JN408112-408113 |
| *Theba* cf. *clausoinflata* "Rock" | FU21 | FU, Degollada de Vinamar (400 m) | N28°04.962’ | W14°20.279’ | 21 | 4 | ZFMK | JN408114-408115 |
| *Theba* cf. *clausoinflata* "Rock" | FU22 | FU, Degollada de Vinamar (500 m) | N28°05.278’ | W14°20.482’ | 22 | 9 | ZFMK | JN408116-408117 |
| *Theba* cf. *clausoinflata* "Rock" | FU23 | FU, Degollada de Vinamar (600 m) | N28°05.809’ | W14°20.999’ | 2 | 10 | ZFMK | JN408118-408119 |
| *Theba* cf. *clausoinflata* "Rock" | FU25 | FU, Pico de la Zarza (800 m) | N28°06.059’ | W14°21.380’ | 2 | 10 | ZFMK | JN408120-408121 |

* = mixed samples (i.e. samples are composed of two species)

^■^ = include samples that were genetically not investigated

^♦^ = ambiguous topological position

^(a)^**Abbreviations** (Locality): FU = Fuerteventura, Spain; GC = Gran Canaria, Spain; LZ = Lanzarote, Spain; P = Portugal.

^(b)^**Abbreviations** (Collection): ZFMK = Zoologisches Forschungsmuseum Alexander Koenig, Bonn, Germany.
