# Supplementary material for: Radiating on Oceanic Islands: Patterns and Processes of Speciation in the Land Snail Genus Theba (Risso 1826)
Source: PLoS One. 2012 Apr 6;7(4):e34339. doi: 10.1371/journal.pone.0034339 (PMC3321021; doi:10.1371/journal.pone.0034339)
Supplement: Table S2 — Summary of AMOVA with and without different hierarchies. Populations with fewer than 5 individuals were excluded from analysis due to small sample size (see Table S1). MOTUs corresponded to results of phylogenetic analyses and the MOTU assignment test. Bold fixation indices were statistically significant. (DOC) [file pone.0034339.s005.doc]

**Table S2.** Summary of AMOVA with and without different hierarchies. Populations with less than 5 individuals were excluded from analysis due to small sample size (see Table S1). MOTUs corresponded to results of phylogenetic analyses and the MOTU assignment test. Bold fixation indices were statistically significant.

| MOTU 1 | MOTU 2 | MOTU 3 | MOTU 4 | MOTU 5 | MOTU 6 | MOTU 7 | MOTU 8 |
| --- | --- | --- | --- | --- | --- | --- | --- |
| *Theba geminata* | *Theba* sp. 2 | *Theba impugnata* | *Theba* sp. 1a | *Theba* sp. 1b | *Theba* sp. 4 | *Theba* cf. *clausoinflata* "Rock" | *Theba* sp. 5 "Sand" |
| Lanzarote | Lanzarote | Lanzarote | Fuerteventura | Fuerteventura | Fuerteventura | Jandía, Fuerteventura | Jandía, Fuerteventura |
| LZ3 |  |  |  |  |  |  |  |
| LZ4 |  |  |  |  |  |  |  |
| LZ6 |  |  |  |  |  |  |  |
| LZ7 | LZ12* |  | FU5 |  |  |  |  |
| LZ8 | LZ15 |  | FU4 | FU7 |  | FU20 |  |
| LZ5* | LZ14 |  | FU27 | FU6 |  | FU21 | FU11 |
| LZ12* | LZ20 |  | FU26 | FU2 |  | FU12 | FU10 |
| LZ21* | LZ18 | LZ5* | LZ23* | FU1 | FU8 | FU22 | FU15 |
| **all 31 populations: no structure** | | | | | **% var** |  | **Fix. indices** |
| among populations | | | | | 24.41 |  | **F_ST_ = 0.24** |
| within populations | | | | | 75.59 |  | ­- |
| **MOTU 1 / 2 / 3 / 4 / 5 / 6 / 7 / 8 (Nb of groups = 8)** | | | | | **% var** |  | **Fix. indices** |
| among groups | | | | | 19.15 |  | **F_CT_ = 0.19** |
| among populations within groups | | | | | 7.13 |  | **F_SC_ = 0.09** |
| among populations | | | | | ­- |  | **F_ST_ = 0.26** |
| within populations | | | | | 73.71 |  | ­- |
| **MOTU 4 (FU5, FU4, FU27, FU26) / LZ23* (Nb of groups = 2)^♦^** | | | | | **% var** |  | **Fix. indices** |
| among groups | | | | | 5.66 |  | F_CT_ = 0.06 |
| among populations within groups | | | | | 7.23 |  | **F_SC_ = 0.08** |
| among populations | | | | | ­- |  | **F_ST_ = 0.13** |
| within populations | | | | | 87.12 |  | ­- |
| **MOTU 4 (FU5, FU4, FU27) / FU26 (Nb of groups = 2)^♦^** | | | | | **% var** |  | **Fix. indices** |
| among groups | | | | | 2.26 |  | F_CT_ = 0.02 |
| among populations within groups | | | | | 6.64 |  | **F_SC_ = 0.07** |
| among populations | | | | | ­- |  | **F_ST_ = 0.09** |
| within populations | | | | | 91.11 |  | ­- |
| **MOTU 1 - 3 (Lanzarote): no structure** | | | | | **% var** |  | **Fix. indices** |
| among populations | | | | | 26.83 |  | **F_ST_ = 0.27** |
| within populations | | | | | 73.17 |  | ­- |
| **MOTU 1 / MOTU 2 - 3 (Nb of groups = 2)** | | | | | **% var** |  | **Fix. indices** |
| among groups | | | | | 28.37 |  | **F_CT_ = 0.28** |
| among populations within groups | | | | | 8.14 |  | **F_SC_ = 0.11** |
| among populations | | | | | ­- |  | **F_ST_ = 0.37** |
| within populations | | | | | 63.49 |  | ­- |
| **MOTU 2 / MOTU 3 (Nb of groups = 2)** | | | | | **% var** |  | **Fix. indices** |
| among groups | | | | | 8.55 |  | F_CT_ = 0.09 |
| among populations within groups | | | | | 9.5 |  | **F_SC_ = 0.10** |
| among populations | | | | | ­ |  | **F_ST_ = 0.18** |
| within populations | | | | | 81.95 |  | **­** |
| **MOTU 4 - 8 (Fuerteventura): no structure** | | | | | **% var** |  | **Fix. indices** |
| among populations | | | | | 16.04 |  | **F_ST_ = 0.16** |
| within populations | | | | | 83.96 |  | ­- |
| **MOTU 4 - 6 / MOTU 7 - 8 (Nb of groups = 2)** | | | | | **% var** |  | **Fix. indices** |
| among groups | | | | | 8.28 |  | **F_CT_ = 0.08** |
| among populations within groups | | | | | 11.09 |  | **F_SC_ = 0.12** |
| among populations | | | | | ­- |  | **F_ST_ = 0.19** |
| within populations | | | | | 80.62 |  | ­- |
| **MOTU 4 / MOTU 5 (Nb of groups = 2)** | | | | | **% var** |  | **Fix. indices** |
| among groups | | | | | 3.67 |  | **F_CT_ = 0.04** |
| among populations within groups | | | | | 8.46 |  | **F_SC_ = 0.09** |
| among populations | | | | | ­- |  | **F_ST_ = 0.12** |
| within populations | | | | | 87.88 |  | ­- |
| **MOTU 7 / MOTU 8 (Nb of groups = 2)** | | | | | **% var** |  | **Fix. indices** |
| among groups | | | | | 14.8 |  | **F_CT_ = 0.15** |
| among populations within groups | | | | | 3.66 |  | **F_SC_ = 0.04** |
| among populations | | | | | ­- |  | **F_ST_ = 0.18** |
| within populations | | | | | 81.54 |  | ­- |
| **MOTU 7 : no structure** | | | | | **% var** |  | **Fix. indices** |
| among populations | | | | | 3.86 |  | **F_ST_ = 0.04** |
| within populations | | | | | 92.49 |  | - |
| **MOTU 8 : no structure** | | | | | **% var** |  | **Fix. indices** |
| among populations | | | | | 4.09 |  | **F_ST_ = 0.05** |
| within populations | | | | | 82.67 |  | - |
| **MOTU 1 - 3 / MOTU 4 - 8 (Nb of groups = 2)** | | | | | **% var** |  | **Fix. indices** |
| among groups | | | | | 8.36 |  | **F_CT_ = 0.08** |
| among populations within groups | | | | | 19.23 |  | **F_SC_ = 0.21** |
| among populations | | | | | ­- |  | **F_ST_ = 0.28** |
| within populations | | | | | 72.41 |  | ­- |
| **MOTU 2 - 3 / MOTU 1 + 4 - 8 (Nb of groups = 2)** | | | | | **% var** |  | **Fix. indices** |
| among groups | | | | | 21.26 |  | **F_CT_ = 0.21** |
| among populations within groups | | | | | 14.46 |  | **F_SC_ = 0.18** |
| among populations | | | | | - |  | **F_ST_ = 0.36** |
| within populations | | | | | 64.28 |  | ­- |

* mixed samples (i.e. samples are composed of two species).

^♦^ hierarchical AMOVA within *Theba* sp. 1a.
