# Supplementary material for: Radiating on Oceanic Islands: Patterns and Processes of Speciation in the Land Snail Genus Theba (Risso 1826)
Source: PLoS One. 2012 Apr 6;7(4):e34339. doi: 10.1371/journal.pone.0034339 (PMC3321021; doi:10.1371/journal.pone.0034339)
Supplement: Table S3 — Canonical variates analysis (CVA) based assignment success. Theba MOTUs along rows, CVA groups along columns. (DOC) [file pone.0034339.s006.doc]

**Table S3.** Canonical variates analysis (CVA) based assignment success. *Theba* MOTUs along rows, CVA groups along columns.

|  | *T. arinagae* | *T. impugnata* | *T. grasseti* | *Theba* sp. 2 | *T. geminata* | *Theba* sp. 1b | *Theba* sp. 1a | *Theba* sp. 4 | *Theba* sp. 5 „Sand“ | *T.* cf. *clausoinflata* „Rock“ | FU23 | FU25 |
| --- | --- | --- | --- | --- | --- | --- | --- | --- | --- | --- | --- | --- |
| 1 | 20  95.2% | 0 | 0 | 0 | 0 | 1 | 0 | 0 | 0 | 0 | 0 | 0 |
| 2 | 0 | 150  79.8% | 16 | 3 | 2 | 4 | 11 | 0 | 2 | 0 | 0 | 0 |
| 3 | 0 | 12 | 147  84.5% | 1 | 0 | 6 | 3 | 1 | 4 | 0 | 0 | 0 |
| 4 | 4 | 0 | 0 | 85  60.7% | 3 | 10 | 4 | 10 | 20 | 2 | 2 | 0 |
| 5 | 5 | 0 | 0 | 0 | 62  83.8% | 1 | 2 | 3 | 1 | 0 | 0 | 0 |
| 6 | 3 | 0 | 0 | 2 | 4 | 28  52.8% | 11 | 5 | 0 | 0 | 0 | 0 |
| 7 | 1 | 1 | 2 | 6 | 3 | 11 | 25  41.0% | 4 | 3 | 5 | 0 | 0 |
| 8 | 0 | 0 | 0 | 2 | 1 | 3 | 1 | 16  51.6% | 5 | 3 | 0 | 0 |
| 9 | 0 | 0 | 0 | 6 | 2 | 0 | 1 | 4 | 49  73.1% | 3 | 2 | 0 |
| 10 | 7 | 0 | 0 | 8 | 1 | 5 | 8 | 10 | 3 | 77  58.3% | 13 | 0 |
| 11 | 0 | 0 | 0 | 0 | 0 | 0 | 0 | 0 | 0 | 2 | 8  80.0% | 0 |
| 12 | 0 | 0 | 0 | 0 | 0 | 0 | 0 | 0 | 0 | 0 | 1 | 9  90.0% |

1 = *T. arinagae*, 2 = *T. impugnata*, 3 = *T. grasseti*, 4 = *Theba* sp. 2, 5 = *T. geminata*, 6 *= Theba sp. 1b,* 7 *= Theba* sp. 1a, 8 = *Theba* sp. 4, 9 = *Theba* sp. 5 „Sand“, 10 = *T.* cf. *clausoinflata* „Rock“, 11 = FU23, 12 = FU25.
