# Supplementary material for: Radiating on Oceanic Islands: Patterns and Processes of Speciation in the Land Snail Genus Theba (Risso 1826)
Source: PLoS One. 2012 Apr 6;7(4):e34339. doi: 10.1371/journal.pone.0034339 (PMC3321021; doi:10.1371/journal.pone.0034339)
Supplement: Table S4 — Results of the simple Mantel test analyses. In the analyses matrices of geographical distance (km) were compared to genetic distances (FST). (DOC) [file pone.0034339.s007.doc]

**Table S4.** Results of simple Mantel test analyses. In the analyses matrices of geographical distance (km) were compared to genetic distances (F_ST_).

| Mantel test: Geographical distance / pairwise F_st_ | | | |
| --- | --- | --- | --- |
| **Island** | **Matrix** | **Mantel`s *r*** | **p-value** |
| Lanzarote + Fuerteventura | all populations | 0.41 | 0.000006 |
|  | *T. geminata* and all populations of Fuerteventura | 0.82 | 0.000001 |
|  | *T. geminata*, *Theba* sp. 1a + 1b, *Theba* sp. 4 and *T.* cf. *clausoinflata* "Rock" | 0.84 | 0.000001 |
| Fuerteventura | all populations of Fuerteventura | 0.64 | 0.000006 |
|  | *Theba* sp. 1a + 1b, *Theba* sp. 4 and *T.* cf. *clausoinflata* "Rock" | 0.88 | 0.000001 |
| Lanzarote | all populations of Lanzarote | 0.27 | 0.013286 |
|  | *T. geminata* | 0.45 | 0.013015 |
|  | *T. impugnata* and *Theba* sp. 2 | 0.75 | 0.006944 |
